# Do saki monkeys possess a grooming claw?

**DOI:** 10.5194/pb-7-19-2020

**Published:** 2020-09-15

**Authors:** Constanze Ohlendorf, Eckhard W. Heymann

**Affiliations:** 1 Soziobiologie/Anthropologie, Georg-August-Universität Göttingen, 37077 Göttingen, Germany; 2 Verhaltensökologie & Soziobiologie, Deutsches Primatenzentrum – Leibniz-Institut für Primatenforschung, 37077 Göttingen, Germany; a current address: Planungsgemeinschaft LaReG, Helmstedter Straße 55A, 38126 Braunschweig, Germany

## Abstract

The presence of a grooming claw on the second toe is a characteristic of Strepsirrhini and tarsiers. There is also some evidence for the presence of a grooming claw in Platyrrhini. Here we report qualitative findings from different species of saki monkeys, genus *Pithecia*, on the presence of modified nails on the second toe. These observations suggest that a grooming claw or a grooming claw-like nail occurs in different *Pithecia* species, but that it does not consistently occur in all individuals.

## 1 Introduction

Grooming claws are a well-known morphological characteristic of Strepsirrhini and tarsiers. Strepsirrhini possess grooming claws on the second toe, tarsiers also on the third toe. Grooming claws differ in shape from other nails and are specifically used for scratching and grooming the fur of the head and neck region (Maiolino et al., 2011; Jolly, 1966). The absence of a grooming claw is regarded as a synapomorphy of Simiae (Anthropoidea), delimiting them from Strepsirrhini and tarsiers (Maiolino et al., 2011).

Grooming claws are a form of unguis, the keratinized structure found on the distal phalanges of the digits in most mammals (Maiolino et al., 2011). In primates, nails (ungulae), claw-like nails (tegulae) and grooming claws can be distinguished. Strepsirrhini possess ungulae, tegulae and a grooming claw on the second pedal digit (Soligo and Müller, 1999; Soligo, 2005). Within Haplorrhini, tarsiers possess ungulae and a grooming claw on the second and third pedal digit; Catarrhini only possess ungulae, whereas within Platyrrhini Callitrichidae also possess tegulae (Maiolino et al., 2011; Soligo and Müller, 1999; Spearman, 1985). The latter are often regarded as claws but differ from the claws of non-primate mammals by the presence of an ungual or apical tuft (Mittra et al., 2007; Soligo and Müller, 1999; Spearman, 1985). These expansions are present on ungular, tegular as well as grooming phalanges (Maiolino et al., 2011). Platyrrhini retain several plesiomorphic primate traits, e.g., the presence of three premolars and the fusion of the ectotympanic ring with the side of the auditory bulla (Fleagle, 2013). Bluntschli (1929) was the first to suggest that Platyrrhini may also retain a grooming claw. He reported modified nails on the second toe of wild individuals of the genera *Aotus*, *Pithecia *and *Saimiri *and described the nails on their second pedal digits as wider and projecting steeper and further above the digital pad. Maiolino et al. (2011) also interpreted the second nail from individuals of the genus *Aotus *as grooming claws. Furthermore, they described tegulae with a grooming-claw-like morphology in *Callicebus* but did not find evidence for a grooming claw in *Pithecia*. They noted that the third (or terminal) phalanges of the second pedal digit of several platyrrhine species were dorsally more canted; i.e., they project dorsally and at a steep angle compared to the third phalanges of the third pedal digit.

Our study aims to contribute to the discussion on the presence of a grooming claw in Platyrrhini. It was stimulated by the depiction of what looks like a grooming claw in a naturalistic engraving (Fig. 1a), observations on a living *Pithecia monachus *(Fig. 1b), and by the contrasting conclusions of Bluntschli (1929) and Maiolino et al. (2011). We observed living individuals, revised museum specimens and reviewed photographic material to examine whether – as suggested by Bluntschli (1929) – a grooming claw might be present in the genus *Pithecia*, the smallest genus of Pitheciinae (Marsh, 2014).

**Figure 1:**
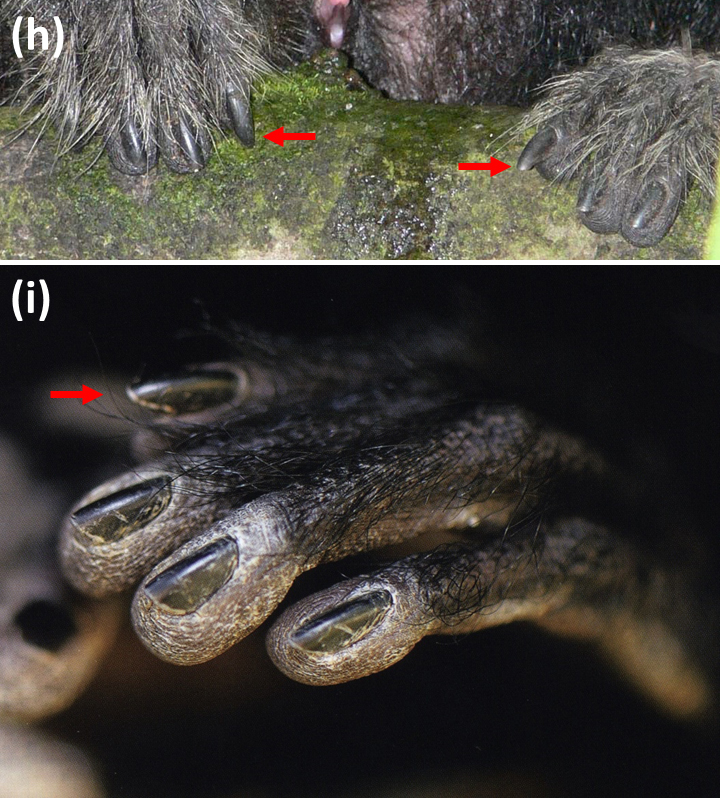


**Figure 1:**
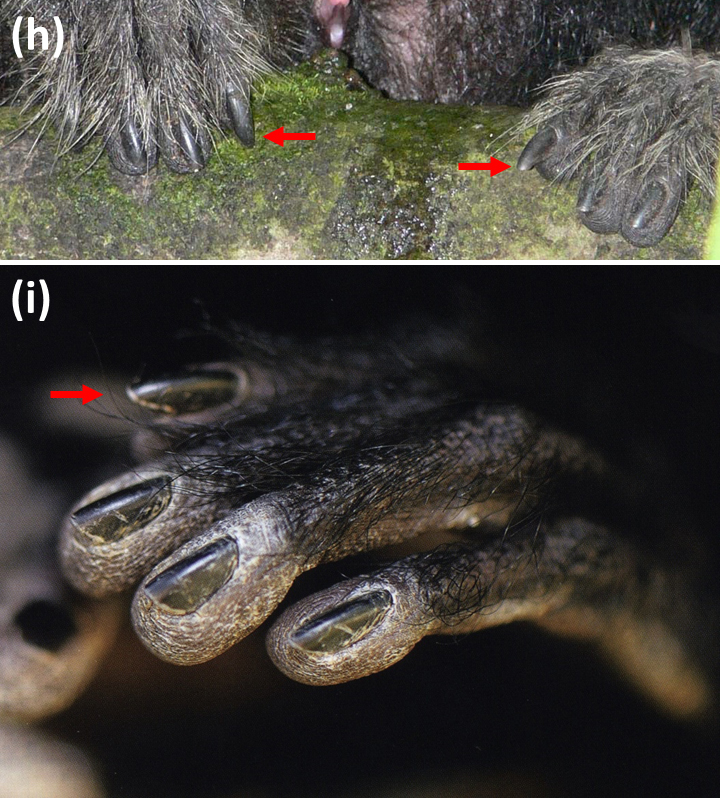
Conspicuous nail appearance on the second pedal digit in individuals of the genus *Pithecia*. **(a)** Engraving of *Pithecia monachus *(photo taken from an engraving in Lydekker (1893–1894) owned by the senior author); **(b)** *Pithecia monachus*, juvenile male (see Bartecki and Heymann, 1987, for information on this in individual), (© Ursula Bartecki); **(c, d)** *Pithecia pithecia*, juvenile male from the Cologne Zoo (© Constanze Ohlendorf); **(e)** *Pithecia monachus *(ZMB_Mam_35984); **(f)** *Pithecia monachus *(ZMB_Mam_46147); **(g)** *Pithecia pithecia *(ZMB_Mam_38456); **(h)** *Pithecia inusta*, juvenile female (© Laura K. Marsh; detail from a photo in Marsh, 2014); **(i)** *Pithecia pithecia*, adult male (© Thomas Marent; detail from a photo in Marent and Jantschke, 2014).

## 2 Methods

### 2.1 Museum specimens

The first author examined 26 specimens in the collections of the Museum für Naturkunde Berlin (ZMB) and the Zoologisches Forschungsmuseum Alexander Koenig in Bonn (ZFMK) (Table S1 in the Supplement). For taxonomic identification, we compared the name given on the label with names given by Marsh (2014) in her revision of the genus *Pithecia* by matching the collection number or the geographic origin where available.

### 2.2 Photographic material

We browsed scientific and popular books and articles that include photos of primates in the library of the Deutsches Primatenzentrum – Leibniz-Institut für Primatenforschung (DPZ).

### 2.3 Observations on living animals (*Pithecia monachus*, *Pithecia pithecia*)

A juvenile male *Pithecia monachus* was temporarily housed at the Estación Biológica Quebrada Blanco (EBQB), a field research site in the north-eastern Peruvian Amazonia. The animal was very tame and could be handled and observed at close range. A juvenile female* P. monachus*, confiscated by the forest police and housed at the Centro de Reproducción y Conservación de Primates no Humanos (CRCP) in Iquitos, could also be examined at close range. For details see Bartecki and Heymann (1987).

A family group of three *Pithecia pithecia* (13-year-old male, 5-year-old female, 1-year-old male) was observed and photographs taken on 27 January 2014 at the Cologne Zoo.

### 2.4 Criteria for considering a grooming claw

Our principal criterion was whether the nail was dorsally canted, i.e., whether the nail formed a larger ankle with the apical pad, since a steep ankle was considered the most noticeable characteristic of grooming claws (Soligo and Müller, 1999; Maiolino et al., 2011). The ankle can be estimated both in museum specimens and in living animals. We considered longitudinal curvature, bilateral compression, pointedness, and projection beyond the apical pad, always in comparison to the nails on the other digits, as additional but weaker criteria (Maiolino et al., 2011). We measured the length and width of nails in museum specimens with a digital caliper from the tip of the nail to the edge of the skin covering the proximal part of the nail. The width-to-length ratio was calculated and used as a proxy to bilateral compression of the nail on the second digit in comparison to the nails on the third and fourth digit. All measurements are available in the Supplement (Table S2).

## 3 Results

The nail on the second digit of the foot of various species of *Pithecia* shows variation in shape and size (Figs. 1, S1, Table S1 in the Supplement).

### 3.1 Living animals

In the juvenile male *P. monachus* the nail on the second digit was strongly dorsally canted, protruded beyond the apical pad and was slightly narrower and more longitudinally curved than the other nails (Fig. 1b). In the juvenile female *P. monachus*, the nail on the second digit was only slightly dorsally canted, did not protrude and was not notably narrower or longitudinally curved.

In all three *P. pithecia* observed at the Cologne Zoo, the nail on the second digit was slightly dorsally canted, protruded strongly beyond the apical pad and was slightly narrower than the other nails, but it was not notably longitudinally curved (Fig. 1c, d).

### 3.2 Museum specimens

In all museum specimens, the nail on the second digit was canted dorsally, but to a varying degree, and in several cases it was also more longitudinally curved than the other nails. The most notable cases are depicted in Fig. 1e–g. While the nail of the second digit seemed to protrude beyond the apical pad in many specimens, this cannot be reliably evaluated, as the soft tissue of the fingertips may have receded in the dried skin, thus giving a false impression.

Measurements of nail length and width and the width-to-length ratio reveal considerable variation (Table S2, Fig. S1). A visual inspection of the specimens' individual profiles for length, width and the width-to-length ratio across digits 2–5 reveals no pattern (Fig. S2).

### 3.3 Photographs

We found two photographs in the literature that were detailed enough to allow for an evaluation. In a juvenile female *Pithecia inusta*, the nail on the second digit protrudes more strongly beyond the apical pad than the other nails; it is neither dorsally canted nor notably longitudinally curved (Fig. 1h). In an adult male *P. pithecia*, the nail on the second digit is dorsally canted and also protrudes beyond the apical pad, different from the nails on the other digits (Fig. 1i).

## 4 Discussion

Our qualitative study provides support for the notion by Bluntschli (1929) of the presence of a grooming claw in *Pithecia*. In several museum specimens and living individuals the nail on the second pedal digit does not conform to the description of a typical ungula, which lies flat on the apical pad and is barely bilaterally compressed (Le Gros Clark, 1936; Maiolino et al., 2011). Rather, compared to a typical ungula, it is generally canted dorsally (albeit to varying degree) and narrower and occasionally pointed, even though less longitudinally curved and pointed than a typical falcula and bilaterally less compressed than a tegula. According to Soligo and Müller (1999) and Maiolino et al. (2011) the most noticeable characteristic of a grooming claw is the steep angle which it forms dorsally with the apical pad. This is also the most obvious characteristic of the several supposed grooming claws shown in Fig. 1. Furthermore, the apical pad is positioned more proximally, which is typical for a grooming claw phalanx (Maiolino et al., 2011) so that the nail is protruding beyond the apical pad. This protrusion could be the result of less abrasion compared to the nails on the other digits, particularly since the functional axis of the foot goes through the fourth digit in platyrrhines (Christoph Soligo, personal communication, 2020).

**Figure 2:**
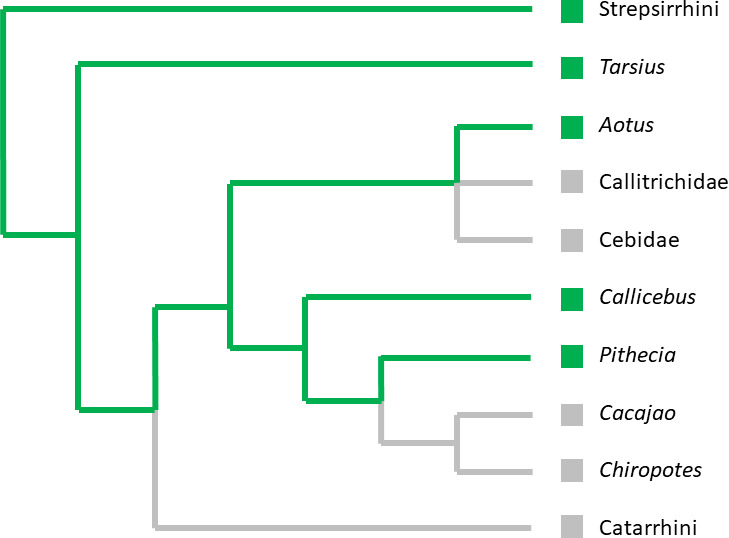
Simplified primate phylogeny showing the presence (
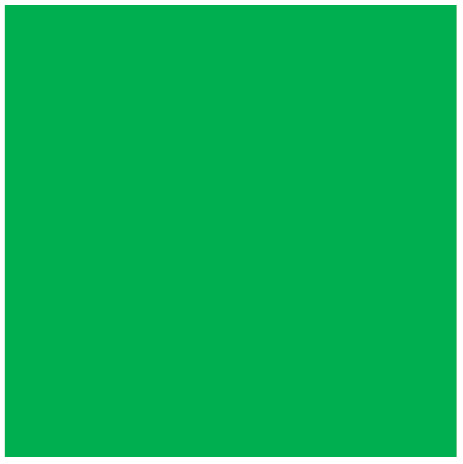
) or absence (
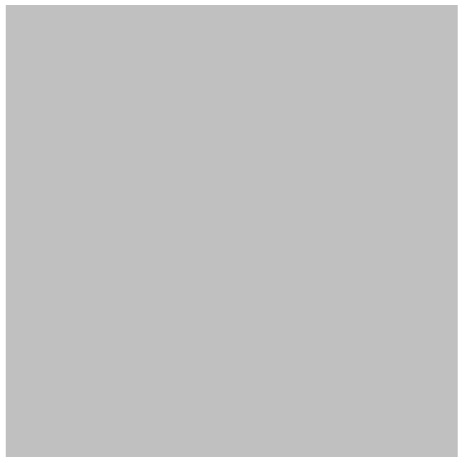
) of a grooming claw in different lineages. We used a polytomy for the clade conformed by *Aotus*, Callitrichidae and Cebidae, as the position of *Aotus* within this clade is contentious (Kay, 2015; Schrago and Seuánez, 2019).

In summary, the nails on the second pedal digits of individuals from the genus *Pithecia *show strong similarities to the grooming claw of strepsirrhines and tarsiers. Nevertheless not all individuals exhibit the same nail morphologies leading to the assumption that a grooming claw or a grooming claw-like nail on the second pedal digit is not a consistent trait within the genus *Pithecia*. If more detailed and quantitative studies reveal that it were indeed a grooming claw, the question then emerges of whether it represents an occasional atavism or an unstable plesiomorphy of the Platyrrhini that has already disappeared not only from several lineages (Atelidae, Callitrichidae, the larger pitheciines) but also from a fraction of those lineages where it still occurs. Mapping the presence/supposed presence of a grooming claw on a primate phylogenetic tree suggests that it might represent a plesiomorphy of Platyrrhini that was lost independently in several lineages (Fig. 2). The assumption of a plesiomorphy predicts that a grooming claw should have been present in the last common ancestor of Platyrrhini and Catarrhini.

It is noticeable that there is only evidence for grooming claws within the smaller genera of Platyrrhini (Bluntschli, 1929; Maiolino et al., 2011). But body size alone cannot be a limiting factor; the indri, *Indri indri*, possesses a grooming claw although it is much larger than all other primates with grooming claws (Mittermeier et al., 2013).

In summary, the answer to the question “Do saki monkeys possess a grooming claw?” is some perhaps do; others do not. We hope that this paper stimulates further research into this question. A more conclusive answer would contribute to the understanding of the evolution and the loss of traits in primate phylogeny.

## Supplement

10.5194/pb-7-19-2020-supplement The supplement related to this article is available online at: https://doi.org/10.5194/pb-7-19-2020-supplement.

## Data Availability

The data on which this paper is based are provided in the Supplement.

## References

[bib1.bib1] Bartecki U, Heymann EW (1987). Über Schweifaffen in Peru. Zeitschrift des Kölner Zoo.

[bib1.bib2] Bluntschli H (1929). Ein eigenartiges an Prosimierbefunde erinnerndes Nagelverhalten am Fuß von platyrhinen Affen. Wilhelm Roux' Archiv für Entwicklungsmechanik der Organismen.

[bib1.bib3] Fleagle JG (2013). Primate adaptation and evolution.

[bib1.bib4] Jolly A (1966). Lemur behavior.

[bib1.bib5] Kay RF (2015). Biogeography in deep time – What do phylogenetics, geology, and paleoclimate tell us about early platyrrhine evolution?. Mol Phylogenet Evol.

[bib1.bib6] Lydekker R (1893). The Royal Society Natural History.

[bib1.bib7] Maiolino S, Boyer D, Rosenberger AL (2011). Morphological correlates of the grooming claw in distal phalanges of platyrrhines and other primates: a preliminary study. Anat Rec.

[bib1.bib8] Marent T, Jantschke F (2014). Affen der Welt, Welt der Affen.

[bib1.bib9] Marsh LK (2014). A taxonomic revision of the saki monkeys, *Pithecia *Desmarest, 1804. Neotropical Primates.

[bib1.bib10] Mittermeier RA, Rylands AB, Wilson DE (2013). Handbook of the Mammals of the World 3. Primates.

[bib1.bib11] Mittra ES, Smith HF, Lemelin P, Jungers WL (2007). Comparative morphometrics of the primate apical tuft. Am J Phys Anthropol.

[bib1.bib12] Schrago CG, Seuánez HN (2019). Large ancestral effective population size explains the difficult phylogenetic placement of owl monkeys. Am J Primatol.

[bib1.bib13] Soligo C (2005). Anatomy of the hand and arm in *Daubentonia madagascariensis*: a functional and phylogenetic outlook. Folia Primatol.

[bib1.bib14] Soligo C, Müller AE (1999). Nails and claws in primate evolution. J Hum Evol.

[bib1.bib15] Spearman RIC (1985). Phylogeny of the nail. J Hum Evol.

