# Supplementary material for: Do saki monkeys possess a grooming claw?
Source: Primate Biol. 2020 Sep 15;7(2):19–23. doi: 10.5194/pb-7-19-2020 (PMC7513581; doi:10.5194/pb-7-19-2020)
Supplement: The supplement related to this article is available online at: https://doi.org/10.5194/pb-7-19-2020-supplement. [file pb-7-19-supplement.zip › pb-7-19-2020-supplement-title-page.pdf]

Supplement of Primate Biol., 7, 19–23, 2020
https://doi.org/10.5194/pb-7-19-2020-supplement
© Author(s) 2020. This work is distributed under
the Creative Commons Attribution 4.0 License.

Primate Biology
Open Access

Supplement of

Do saki monkeys possess a grooming claw?

Constanze Ohlendorf and Eckhard W. Heymann

Correspondence to: Eckhard W. Heymann (eheyman@gwdg.de)

- pb-7-19-2020-supplement-title-page.pdf
- Supplementary Figures S1 and S2.pdf
- Supplementary Table S1. Museum specimens.pdf
- Supplementary Table S2. Nail measurements.xlsx

The copyright of individual parts of the supplement might differ from the CC BY 4.0 License.
