# Supplementary material for: Do saki monkeys possess a grooming claw?
Source: Primate Biol. 2020 Sep 15;7(2):19–23. doi: 10.5194/pb-7-19-2020 (PMC7513581; doi:10.5194/pb-7-19-2020)

Supplementary figures

Constanze Ohlendorf & Eckhard W. Heymann

Do saki monkeys possess a grooming claw?

Figure S1.

Mean length, width and width/length ratio of nails on the right (R) and left (L) foot of *Pithecia monachus* (blue) and *Pithecia pithecia* (green). Whiskers are ±95% confidence intervals.

(a) Length

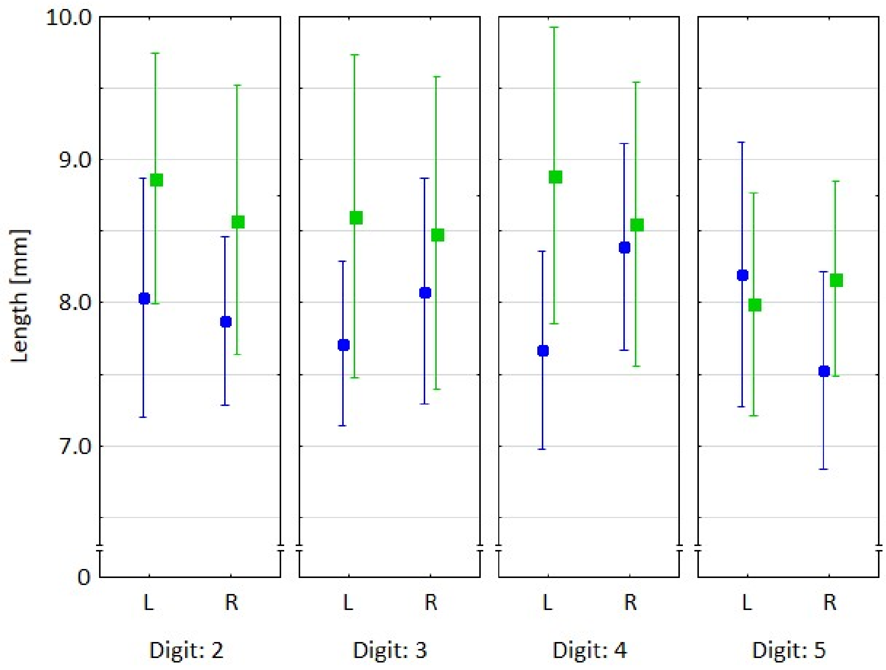

(b) Width

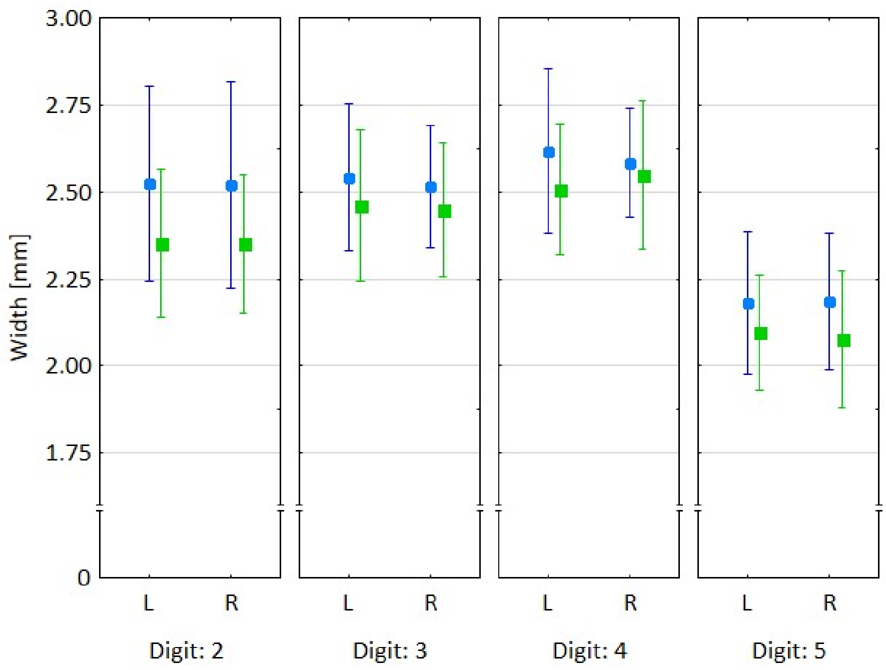

(c) Width/length ratio

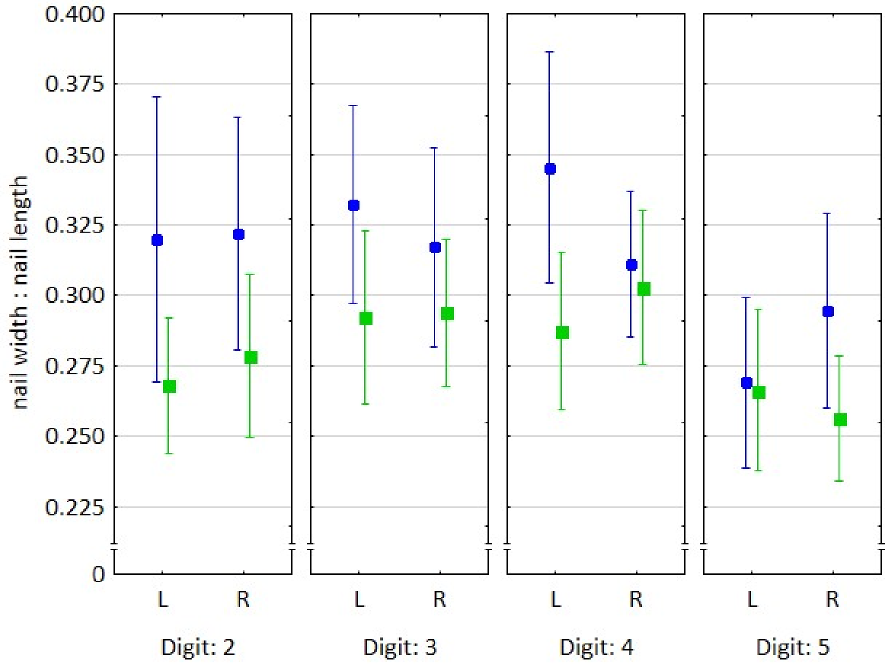

Figure S2.

Individual profiles for length and width of nails and for the width/length ratio.

Blue: *Pithecia monachus*, green: *Pithecia pithecia*

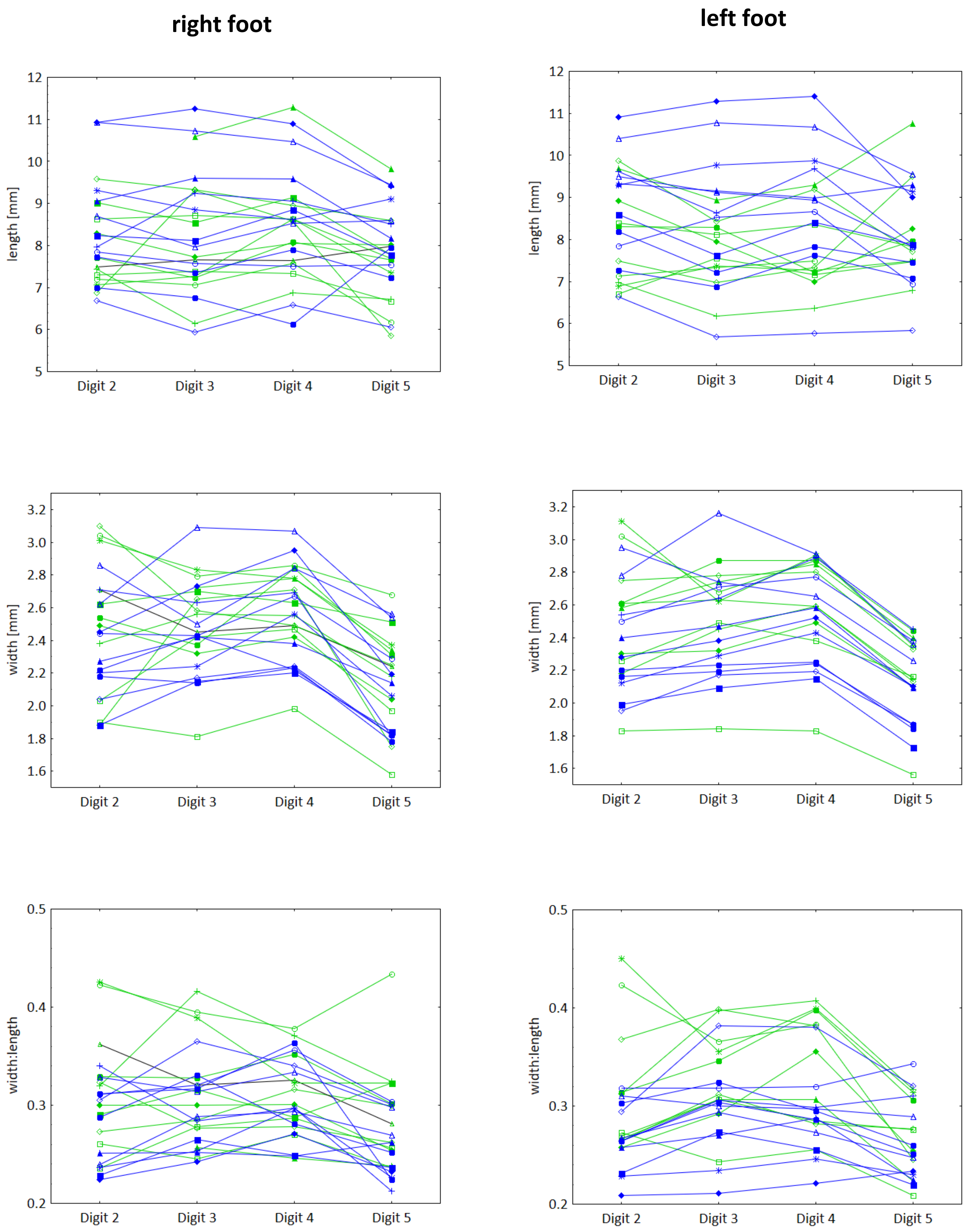

Supplement: The supplement related to this article is available online at: https://doi.org/10.5194/pb-7-19-2020-supplement. [file pb-7-19-supplement.zip › Supplementary Figures S1 and S2.pdf]
