# Supplementary material for: Do saki monkeys possess a grooming claw?
Source: Primate Biol. 2020 Sep 15;7(2):19–23. doi: 10.5194/pb-7-19-2020 (PMC7513581; doi:10.5194/pb-7-19-2020)
Supplement: The supplement related to this article is available online at: https://doi.org/10.5194/pb-7-19-2020-supplement. [file pb-7-19-supplement.zip › Supplementary Table S1. Museum specimens.pdf]

Supplementary information.

Table S1. Specimens examined at the Zoologisches Forschungsmuseum Alexander Koenig, Bonn (ZFMK) and the Museum für Naturkunde Berlin (ZMB)

Information for species, sex, origin and year as per label on the specimen except where noted otherwise.

**Collection number**	**Species**	**Sex**	**Origin**	**Year**	**Type of material**
ZFMK_Mam_75.108	*Pithecia irrorata*	female	Duisburg Zoo	1975	skin
ZFMK_Mam_81.1818	*Pithecia aequatorialis*	male	Cologne Zoo	1981	skin
ZMB_Mam_245	*Pithecia pithecia*	male	Guiana	n.a.	mounted
ZMB_Mam_246	*Pithecia pithecia*	female	Guiana	n.a.	skin
ZMB_Mam_5653	*Pithecia* sp.	unknown	Sarayaca	n.a.	mounted
ZMB_Mam_5918	*Pithecia monachus*	male	Ecuador	n.a.	skin
ZMB_Mam_11890	*Pithecia monachus*	female	Zoologischer Garten	1899	skin
ZMB_Mam_16495	*Pithecia monachus*	female	Zoologischer Garten	n.a.	skin
ZMB_Mam_33939	*Pithecia monachus*	female	Canabouca (Amazon region)	1925	skin
ZMB_Mam_35308	*Pithecia monachus*	male	Ayapua (Rio Murus*)	1925	skin
ZMB_Mam_35461	*Pithecia monachus*	female	Canabouca (Amazon region)	1925	skin
ZMB_Mam_35768	*Pithecia monachus*	male	Ayapua (Rio Murus*)	1926	skin
ZMB_Mam_35769	*Pithecia monachus*	male	Ayapua (Rio Murus*)	1926	skin
ZMB_Mam_35984	*Pithecia monachus*	male	unknown	1925	skin
ZMB_Mam_38456	*Pithecia pithecia*	female	Manacapuru (Amazonas)[§]	1924	skin
ZMB_Mam_38457	*Pithecia pithecia*	female	Manacapuru (Amazonas)[§]	1924	skin
ZMB_Mam_38461	*Pithecia pithecia*	male	Manacapuru (Amazonas)[§]	1924	skin
ZMB_Mam_38462	*Pithecia pithecia*	male	Manacapuru (Amazonas)[§]	n.a.	skin
ZMB_Mam_38463	*Pithecia pithecia*	male	Manacapuru (Amazonas)[§]	1924	skin
ZMB_Mam_39460	*Pithecia pithecia*	female	Tapuhy (Rio Jary)	1936	skin
ZMB_Mam_39753	*Pithecia pithecia*	unknown (juvenile)	Brasilien	1937	skin
ZMB_Mam_46146	*Pithecia monachus*	female	Lago do Ayapua (Rio Purus)[#]	1932	skin
ZMB_Mam_46147	*Pithecia monachus*	female	middle course of Rio Manacapuru (Amazonas)[§]	1929	skin
ZMB_Mam_71578	*Pithecia pithecia*	male	Guiana	n.a.	mounted
ZMB_Mam_91313	*Pithecia pithecia*	male	Rio Ica	1930	skin

ZMB_Mam_91314	*Pithecia monachus*	male	Canabouca (Amazon region)	1927	skin

* likely to be a misspelling of Rio Purus

[#] probably Lago Aiapuá on the left bank of the lower Rio Purus

[§] a river draining into the left bank of the Amazon

For three other skins (ZFMK_Mam_71.097, *Pithecia pithecia*, male, Cologne Zoo, 1971; ZFMK_MAM_ 87.730; *Pithecia inusta*, female, Panguana, 1973; ZFMK_MAM_2012.009, *Pithecia pithecia*, male, Frankfurt Zoo, 2008) the nails were separated from the skin or the skin did not have nails.
